# The Fate of Bacterial Spores in the Animal Body

**Published:** 1918-03

**Authors:** 


***The Fate of Bacterial Spores in the Animal Body.*** By STEWARD A. KOSER and JOHN B. McCLELLAND of the Sheffield Laboratory of Bacteriology and Hygiene, Yale University. *The Journal of Medical Research*, Nov., 1917.

The authors report the results of experiments upon white mice to determine the location and the period of the persistence of the spores of various organisms in the animal body. They find that the spores of B. tetani, B. putrificus, B. anthracis symptomatici, and B. œdematis maligni are capable of resisting the destructive influence of the body tissues, and that they may be transported from the site of inoculation to different organs, where they may remain latent for varying periods of time. Tetanus spores in some cases persisted at the site of inoculation for four months and in the liver and spleen for two months. The spores of the B. putrificus persisted in the liver and spleen as well as at the site of inoculation for a period of four months.

The authors state that they can give no satisfactory explanation of the persistence of viable spores of B. tetani and the putrefying organisms in the different tissues. Aerobes could be recovered from tissues away from the site of inoculation in only a few instances.

They believe that their observations may have a practical value in the treatment of wound infection, gangrene, and so-called blood-poisoning. They think that similar studies of the gas bacillus may furnish data of much importance.

